# Molecular Epidemiology and Spatial Distribution of *Toxocara canis* and *Toxocara cati* in Avian Paratenic Hosts: A One Health Perspective From Northeastern Iran

**DOI:** 10.1002/vms3.71310

**Published:** 2026-11-01

**Authors:** Mohammad‐Reza Rezaiemanesh, Rasoul Alimi, Abdolmajid Gholizadeh, Seyed‐Hossein Hejazi, Mohammad‐Ali Mohaghegh

**Affiliations:** ^1^ Department of Laboratory Sciences School of Paramedical Sciences Torbat Heydariyeh University of Medical Sciences Torbat Heydariyeh Iran; ^2^ Department of Medical Parasitology and Mycology School of Medicine Isfahan University of Medical Sciences Isfahan Iran; ^3^ Department of Epidemiology and Biostatistics School of Health Torbat Heydariyeh University of Medical Sciences Torbat Heydariyeh Iran; ^4^ Department of Environmental Health Engineering School of Health Torbat Heydariyeh University of Medical Sciences Torbat Heydariyeh Iran; ^5^ Public Health Research Center Torbat Heydariyeh University of Medical Sciences Torbat Heydariyeh Iran

**Keywords:** bird, Iran, PCR, spatial analysis, *Toxocara canis*, *Toxocara cati*

## Abstract

**Background:**

*Toxocara canis* and *Toxocara cati* are zoonotic nematodes, and birds may contribute to their transmission as paratenic hosts. Data on their molecular occurrence in avian hosts remain limited in northeastern Iran.

**Objective:**

This study investigated the molecular detection and spatial distribution of *T. canis* and *T. cati* in synanthropic and domestic birds from Razavi Khorasan Province.

**Methods:**

In this cross‐sectional study, 300 tissue samples (brain, eye and liver) were collected from 100 birds: sparrows (*n = *60), pigeons (*n = *21) and chickens (*n = *19). Detection and species identification were performed using multiplex nested PCR targeting ITS regions. Spatial patterns were assessed using kernel density estimation and Global Moran's I in ArcGIS 10.8. Associated factors were analysed by multivariable mixed‐effects logistic regression.

**Results:**

*Toxocara* spp. DNA was detected in 51/300 tissues (17.0%) and 35/100 birds (35.0%). Tissue‐level positivity was highest in sparrows (23.3%), followed by chickens (8.8%) and pigeons (6.3%). In sparrows, detection differed among tissues (*p = *0.026), with higher positivity in eye and liver. *T. canis* predominated (68.6%), followed by mixed infections (17.7%) and *T. cati* (13.7%). Bird species and tissue type were independently associated with PCR positivity. Kernel Density Estimation suggested localised southern and southeastern hotspots, whereas Global Moran's I showed a non‐significant random spatial pattern.

**Conclusions:**

Synanthropic and domestic birds, particularly sparrows, may act as paratenic hosts for *Toxocara* spp. in northeastern Iran. Higher detection in eye and liver tissues suggests zoonotic relevance, especially through undercooked poultry giblets; however, DNA detection does not confirm larval viability.

## Graphical abstract

Molecular surveillance in northeastern Iran reveals birds, particularly sparrows, as significant paratenic hosts for *Toxocara* species. With high DNA detection in liver and eye tissues, our findings highlight a One Health zoonotic risk via the consumption of undercooked poultry, emphasising the need for targeted public health awareness and monitoring.

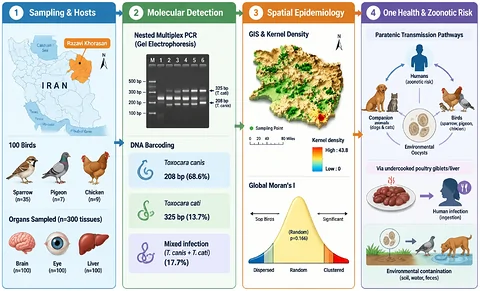

## 1 Introduction

*Toxocara canis* and *Toxocara cati*, the common ascarid nematodes of dogs and cats, are among the most important zoonotic helminths worldwide (Mohaghegh et al. 2018; Rezaiemanesh et al. 2019). Their eggs are shed into the environment through the faeces of infected definitive hosts, where they embryonate and become infective under favourable conditions. Humans and a wide range of vertebrate hosts may acquire infection through ingestion of embryonated eggs or through consumption of paratenic hosts harbouring somatic larvae (Choi et al. 2012; Wu and Bowman 2020). Although the public health significance of toxocariasis has been widely recognised, transmission dynamics involving wildlife and free‐ranging domestic animals remain insufficiently characterised in many endemic settings (Ding et al. 2026; Dutra et al. 2014).

Birds are increasingly acknowledged as epidemiologically relevant paratenic hosts in the life cycle of *Toxocara* spp. After ingestion of infective eggs from contaminated soil, water or food sources, larvae may migrate through the host and persist within tissues such as the liver, eyes, brain and musculature (Healy et al. 2022; Holland 2023). Because many native and synanthropic bird species forage directly on the ground and live in close contact with human settlements, domestic animals and contaminated environments, they may serve as important sentinels of environmental contamination as well as potential links in parasite transmission (Dutra et al. 2014; Keegan et al. 2025). In addition, predation or scavenging of infected birds by dogs and cats may facilitate maintenance of the parasite cycle and contribute to ongoing circulation in peri‐domestic ecosystems (Lopez‐Alamillo et al. 2025).

Recent studies have highlighted the growing value of molecular techniques for detecting *Toxocara* DNA in animal tissues, particularly in hosts in which direct parasitological identification is difficult or impossible (Michelutti et al. 2021; Mohaghegh et al. 2021). PCR‐based methods not only improve diagnostic sensitivity and specificity but also enable species‐level discrimination between *T. canis* and *T. cati*, which is essential for understanding host–parasite relationships, ecological transmission pathways and regional epidemiology (Pal et al. 2023; Rezaiemanesh et al. 2019). Furthermore, integrating molecular findings with spatial analytical tools may provide additional insight into the geographic distribution of infection and help identify areas with elevated transmission intensity (Santos et al. 2009; Shokri et al. 2022).

Despite these advances, a significant gap remains in understanding the tissue‐specific distribution of *Toxocara* larvae in avian paratenic hosts, particularly with respect to potential differences between *T. canis* and *T. cati* (Wu and Bowman 2020). Although larval migration to visceral, ocular and neural tissues has been reported, whether these species exhibit distinct tissue tropisms in naturally exposed birds remains unresolved (Bowman 2020). Addressing this question is epidemiologically important, as species‐specific localisation may shape larval persistence, host infectivity to predators, molecular detectability and the interpretation of transmission dynamics in endemic settings.

Although increasing international interest in zoonotic helminths has expanded the global evidence base, data on avian toxocariasis in Iran remain limited, particularly in northeastern regions where environmental contamination with *Toxocara* eggs, together with close interactions among birds, stray carnivores and human populations, may facilitate transmission. Razavi Khorasan Province constitutes a particularly pertinent setting for such investigation due to its ecological heterogeneity, extensive rural and peri‐urban interfaces, and environmental conditions conducive to the persistence of parasite eggs.

Accordingly, the present study aimed to detect *T. canis* and *T. cati* using molecular methods in native birds from Razavi Khorasan Province, northeastern Iran, using multiplex nested PCR, and to characterise the epidemiological distribution of infection by bird species, tissue type and spatial occurrence. Particular emphasis was placed on evaluating tissue‐specific detection patterns of the two *Toxocara* species to address a key gap in current knowledge regarding larval tropism in avian paratenic hosts. By determining whether species‐level differences exist in tissue distribution, this study seeks to refine understanding of the ecological role of birds in the local transmission cycle of *Toxocara* spp. and to provide epidemiologically relevant evidence for surveillance and risk assessment.

## 2 Materials and Methods

### 2.1 Study Design and Sampling Framework

This cross‐sectional opportunistic survey was conducted in 2022 in Razavi Khorasan Province, northeastern Iran, to investigate the molecular occurrence of *T. canis* and *T. cati* in avian paratenic hosts.

To achieve broad geographic coverage, the province was stratified into five zones (north, south, east, west and central) based on administrative and ecological characteristics. From each zone, two counties were randomly selected. Within the selected counties, bird carcasses were collected opportunistically based on availability during the study period. Sampling relied on freshly dead birds encountered in urban, peri‑urban and rural environments. No live birds were captured, handled or euthanised for research purposes. A total of 100 birds were included in the study. The sample size was determined pragmatically based on logistical feasibility and comparable regional studies investigating *Toxocara* infection in wildlife hosts. All specimens were handled using disposable gloves and sterile instruments to minimise cross‑contamination. Each carcass was individually labelled, and the geographic coordinates of sampling locations were recorded using a handheld GPS device for subsequent spatial analysis.

### 2.2 Bird Specimens and Tissue Sampling

A total of 100 birds were examined in this study, including sparrows (family Passeridae, *n = *60), pigeons (family Columbidae, *n = *21) and chickens (*Gallus gallus* domesticus, *n = *19).

Sparrows and pigeons consisted of carcasses obtained during field sampling in the study areas.

Native chicken samples were obtained after routine slaughter for human consumption and were not killed for research purposes. During necropsy, brain, liver and eye tissues were aseptically collected from each bird, resulting in a total of 300 tissue samples. Each tissue specimen was processed individually to prevent cross‑contamination. Tissues were homogenised using a sterile glass homogeniser and transferred into sterile microtubes for subsequent DNA extraction.

### 2.3 DNA Extraction

Genomic DNA was extracted from tissue samples using the FavorPrep tissue genomic DNA extraction mini kit (Favorgen Biotech Corporation, Taiwan) according to the manufacturer's instructions. DNA was eluted in 50 µL of elution buffer and stored at −20°C until PCR analysis. A negative extraction control was included in each batch.

### 2.4 Nested Multiplex PCR for Detection of *Toxocara* Species

A nested multiplex PCR targeting the ITS1/ITS2 rDNA region was performed according to the protocol of Wang et al. (2018), using species‐specific primers to amplify 208‐bp and 325‐bp fragments for *T. canis* and *T. cati*, respectively (Figure 1).

**FIGURE 1:**
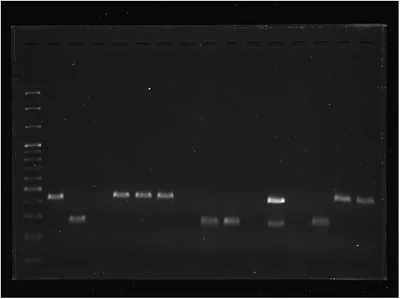
Gel electrophoresis of nested multiplex PCR for molecular detection of *Toxocara* spp. in avian tissue samples. Lane 1, 100 bp DNA ladder; Lane 2, *Toxocara cati*‐positive control (325 bp band); Lane 3, *Toxocara canis*‐positive control (208 bp band); Lane 4, negative control; Lanes 5–7 and 15–16, *Toxocara cati*–positive samples; Lanes 9, 10 and 14, *Toxocara canis*–positive samples; Lane 12, mixed infection.

PCR reactions were carried out in a 25 µL volume containing 12.5 µL of 2× Taq DNA Polymerase Master Mix (Ampliqon, Denmark), 0.3 µM of each primer and 1 µL of template DNA. Thermal cycling consisted of an initial denaturation at 94°C for 2 min, followed by 40 cycles of 94°C for 15 s, 52°C for 30 s and 68°C for 1 min, with a final extension at 68°C for 2 min. Positive controls (confirmed *T. canis* and *T. cati* DNA) and a no‑template control were included in each run. DNA extraction, PCR setup and post‑PCR analysis were conducted in separate laboratory areas to prevent contamination.

### 2.5 Sequence Confirmation

To verify the accuracy of the nested multiplex PCR results, six randomly selected positive PCR products from tissue samples were subjected to sequencing. The obtained sequences were analysed using the BLAST algorithm and compared with reference sequences available in the GenBank database to confirm species identity.

### 2.6 Statistical Analysis

All statistical analyses were performed using IBM SPSS Statistics software version 26 (IBM Corp., Armonk, NY, USA). Descriptive statistics were calculated to summarise the frequency and percentage of PCR‑positive and PCR‑negative samples according to bird species and tissue type. Associations between categorical variables were evaluated using contingency tables and Fisher's exact test due to the presence of small expected cell counts. To identify independent predictors of *Toxocara* PCR positivity, a multivariable binary logistic regression model was applied. The PCR result (0 = negative, 1 = positive) was considered the dependent variable, while bird species and tissue types were included as categorical independent variables. In the regression model, chickens and liver tissue were defined as reference categories. Adjusted odds ratios (OR) with 95% confidence intervals (95% CI) were calculated. A *p*‑value < 0.05 was considered statistically significant. Because three tissues were sampled from each bird, a mixed‐effects logistic regression model with bird identity as a random intercept was used to account for within‐bird correlation.

### 2.7 Spatial Analysis

Spatial analyses were performed using ArcGIS Desktop version 10.8 (ESRI, Redlands, CA, USA). The geographic coordinates of sampling locations where birds were collected were mapped in ArcMap to visualise the spatial distribution of *Toxocara* PCR‑positive samples across Razavi Khorasan Province. Kernel density estimation (KDE) was applied to the locations of PCR‑positive samples to identify areas with higher local densities of infection. The output cell size and search radius were selected according to the spatial distribution of sampling points and the spatial scale of the study area. Density values were expressed as the estimated number of positive cases per square kilometre. Global spatial autocorrelation was evaluated using Moran's I statistic to determine whether the spatial distribution of positive samples deviated from a random pattern. Moran's I was calculated based on the spatial coordinates of PCR‑positive sampling locations using an inverse‑distance spatial weighting scheme. Statistical significance was assessed using permutation testing to generate corresponding z‑scores and *p*‑values, allowing classification of the spatial pattern as clustered, dispersed or random.

## 3 Results

### 3.1 General Prevalence and Tissue Distribution

Molecular analysis was performed on 300 tissue samples obtained from 100 individual birds, comprising sparrows (Passeridae, *n = *60), pigeons (Columbidae, *n = *21) and domestic chickens (*Gallus gallus* domesticus, *n = *19). At the host level, 35 out of 100 examined birds (35.0%; 95% CI: 24.6–44.7) were positive for *Toxocara* spp. DNA in at least one tissue. At the tissue level, the overall positivity rate was 17.0% (51/300; 95% CI: 12.7–21.3). Significant heterogeneity in infection rates was observed across different tissue types (*p = *0.012). The highest detection rates were recorded in the eye (23/100, 23.0%) and liver (20/100, 20.0%), while the brain (8/100, 8.0%) exhibited the lowest frequency of parasite DNA detection (Table 1).

**TABLE 1:** Prevalence of *Toxocara* spp. DNA in brain, eye and liver tissues of examined avian species.

Avian species	Tissue type	Positive/total	Prevalence % [95% CI]	*p*‐value
Sparrow (*n = *60)	Brain	7/60	11.7 [5.8–22.2]	0.026^*^
	Eye	19/60	31.7 [21.3–44.2]	
	Liver	16/60	26.7 [17.1–39.0]	
	Tissue‐level positivity	42/180	23.3 [17.1–29.5]	
	Bird‐level positivity	28/60	46.7 [34.6–59.1]	
Pigeon (*n = *21)	Brain	0/21	0.0 [0.0–15.5]	0.344
	Eye	2/21	9.5 [2.7–28.9]	
	Liver	2/21	9.5 [2.7–28.9]	
	Tissue‐level positivity	4/63	6.3 [0.3–12.3]	
	Bird‐level positivity	3/21	14.3 [5.0–34.6]	
Chicken (*n = *19)	Brain	1/19	5.3 [0.9–24.6]	0.803
	Eye	2/19	10.5 [2.9–31.4]	
	Liver	2/19	10.5 [2.9–31.4]	
	Tissue‐level positivity	5/57	(8.8) [1.5–16.1]	
	Bird‐level positivity	4/19	21.1 [8.5–43.3]	
Total	All samples	51/300	17.0 [12.7–21.3]	0.012^*^
	All birds	35/100	35.0 [24.6–44.7]	

*Note: p*‐values represent comparisons among tissue types within each avian species using Fisher's exact test.

*Significant at *P* < 0.05.

### 3.2 Host‐Specific and Tissue‐Specific Positivity

The prevalence of *Toxocara* DNA varied significantly among the studied avian hosts. Sparrows exhibited the highest bird‐level prevalence at 46.7% (28/60), followed by chickens (21.1%, 4/19) and pigeons (14.3%, 3/21). At the tissue level, sparrows also showed the highest positivity rate (23.3%, 42/180). Within this species, a significant difference was observed between organs (*p = *0.026); specifically, the eye (31.7%) and liver (26.7%) were more frequently affected than the brain (11.7%). In contrast, although the brain remained the organ with the lowest detection rate in pigeons (0%) and chickens (5.3%), no statistically significant differences were found between tissue types within these two species (*p < *0.05) (Table 1; Figure 2).

**FIGURE 2:**
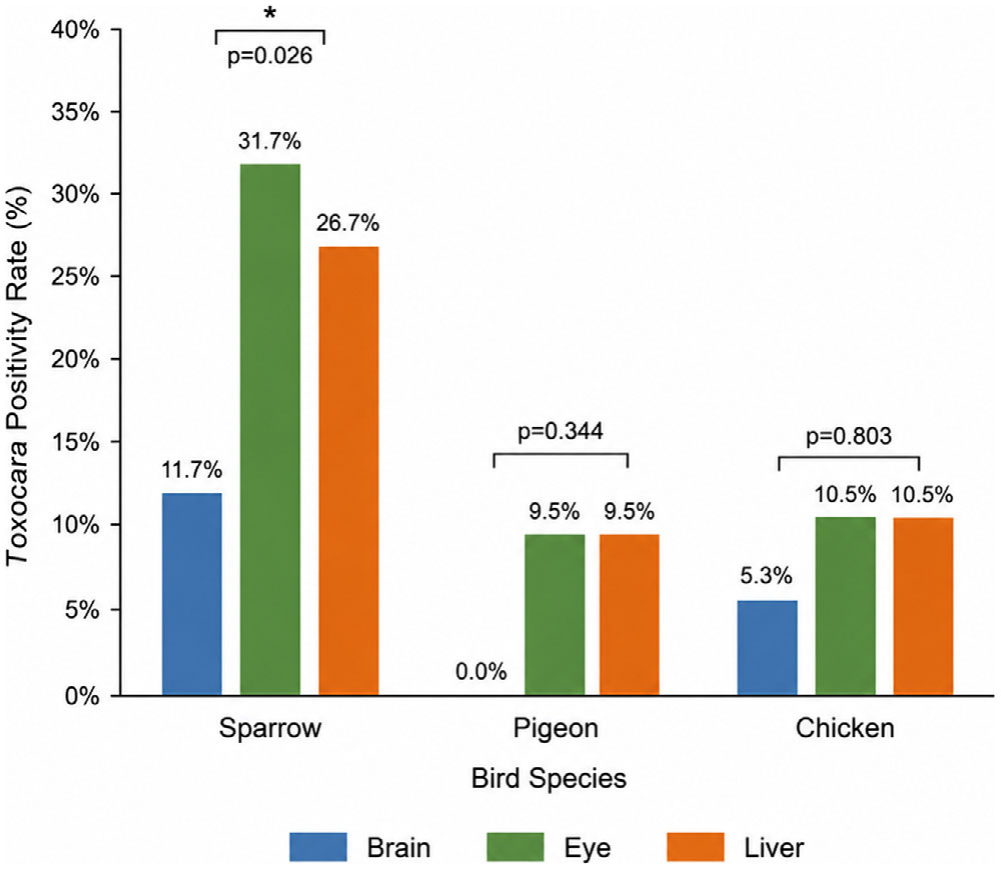
Distribution of *Toxocara* spp. detection across examined tissues (brain, eye and liver) among synanthropic and domestic birds, based on nested multiplex PCR results.

### 3.3 Predictors of PCR Positivity

A multivariable mixed‑effects logistic regression model was used to evaluate factors associated with *Toxocara* PCR positivity while accounting for clustering of tissue samples within individual birds. Bird identity was included as a random intercept to control for within‑bird correlation among the three examined tissues.

Bird species was significantly associated with PCR positivity. Compared with chickens (reference category), sparrows had significantly higher odds of infection (adjusted OR = 3.32, 95% CI: 1.02–10.79, *p = *0.045). In contrast, pigeons did not show a statistically significant difference in infection odds compared with chickens (adjusted OR = 0.73, 95% CI: 0.18–2.94, *p = *0.654). Regarding tissue distribution, the probability of detecting *Toxocara* DNA in the brain was significantly lower than in the liver (adjusted OR = 0.31, 95% CI: 0.12–0.78, *p* = 0.013). No significant difference was observed between the eye and the liver (adjusted OR = 1.34, 95% CI: 0.48–3.73, *p* = 0.581).

The random intercept variance for individual birds was estimated at 1.168 (*p* = 0.021), indicating significant variability in infection probability among birds beyond the fixed effects included in the model (Table 2).

### 3.4 Molecular Identification of *Toxocara* Species

Among the 51 PCR‐positive samples, *Toxocara* canis was the predominant species (35/51, 68.6%), followed by mixed infections of *T. canis* and T. *cati* (9/51, 17.7%) and *T. cati* alone (7/51, 13.7%). While sparrows and pigeons were primarily infected with *T. canis* (71.4% and 75.0%, respectively), chickens showed a higher descriptive proportion of mixed infections (40.0%). However, the distribution of *Toxocara* species did not differ significantly across the three avian hosts (Fisher's Exact *p* = 0.671) (Table 3; Figure 3).

**TABLE 2:** Multivariable mixed‑effects logistic regression analysis of factors associated with *Toxocara* PCR positivity in avian tissues.

Variables	Categories	Adjusted odds ratio (95% CI)	*p*‐value
Avian type	Sparrow	3.32 (1.02–10.79)	0.045^*^
	Pigeon	0.73 (0.18–2.94)	0.654
	Chicken	Reference	—
Tissue type	Brain	0.31(0.12–0.78)	0.013^*^
	Eye	1.34 (0.48–3.73)	0.581
	Liver	Reference	—
Random effect	Intercept (Variance)	1.168	0.021^*^

* Significant at *p* < 0.05.

**TABLE 3:** Distribution of *Toxocara* species identified in PCR‐positive samples.

Avian host	*T. canis* n (%)	*T. cati* n (%)	Mixed infection^*^ n (%)	Total positive
Sparrow	30 (71.4%)	6 (14.3%)	6 (14.3%)	42
Pigeon	3 (75.0%)	0 (0.0%)	1 (25.0%)	4
Chicken	2 (40.0%)	1 (20.0%)	2 (40.0%)	5
Total	35 (68.6%)	7 (13.7%)	9 (17.7%)	51

^Note:^ Comparison of species distribution across hosts was not statistically significant (Fisher's exact test, *p = *0.671).

* Mixed infection was defined as co‐detection of *T. canis* and *T. cati* DNA in the same tissue specimen.

**FIGURE 3:**
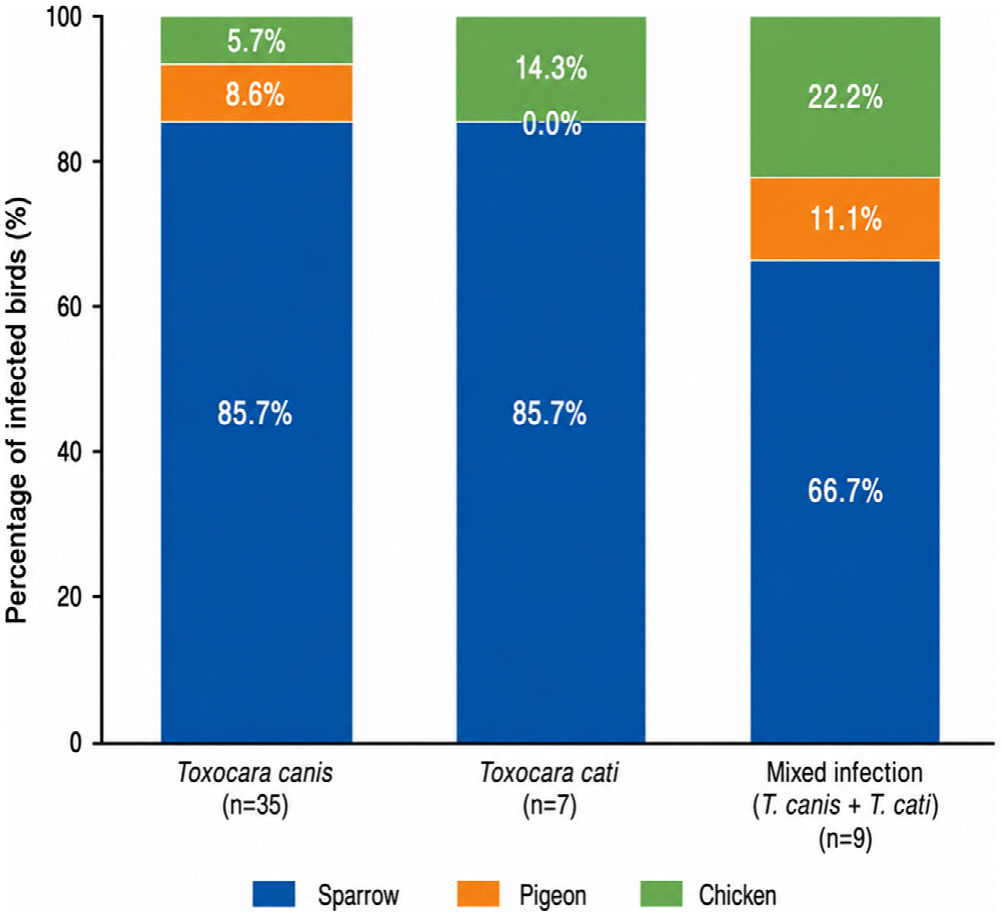
Distribution of *T*. *canis*, *T. cati* and mixed infections among PCR‐positive tissue samples (*n = *51) from sparrows, pigeons and chickens.

### 3.5 Spatial Distribution and Autocorrelation Analysis

KDE maps visualised the spatial intensity of positive samples, revealing localised hotspots in the southern and southeastern regions of Razavi Khorasan Province (Figure 4). Despite these local variations, Global Moran's I analysis yielded a value of 0.019 with a *z*‐score of 1.382 (*p* = 0.166), indicating that the distribution of *Toxocara* DNA across the province does not follow a significant global clustering pattern and is statistically consistent with a random spatial distribution (Figure 5).

**FIGURE 4:**
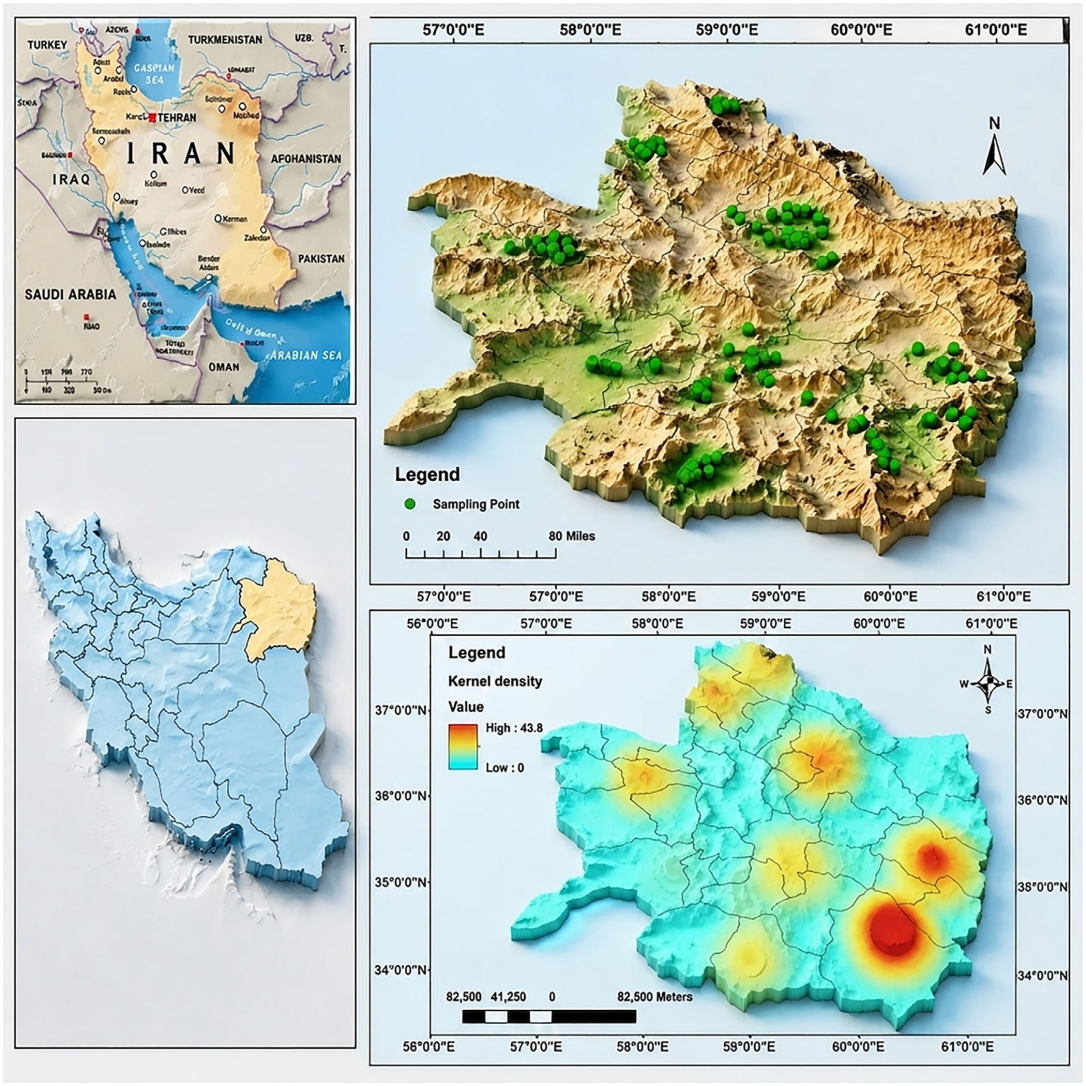
Spatial distribution of sampled birds and kernel density estimation (KDE) map highlighting spatial hotspots of *Toxocara* spp. PCR‑positive samples in Razavi Khorasan Province, Iran.

**FIGURE 5:**
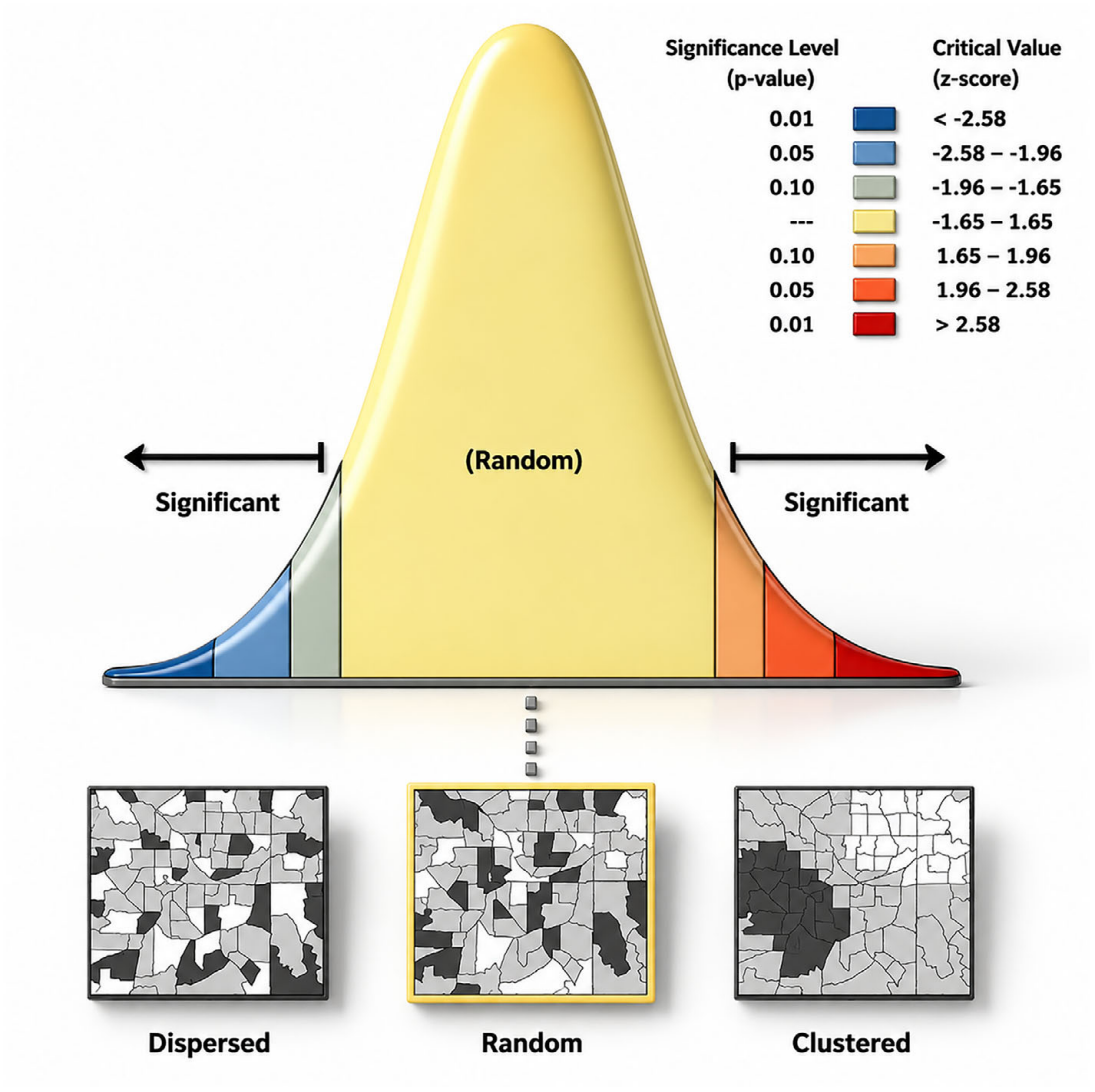
Graphic representation of Global Moran's I significance levels and spatial patterns.

## 4 Discussion

The present study provides molecular evidence that free‐ranging birds in Razavi Khorasan Province act as avian paratenic hosts for *Toxocara* spp., with parasite DNA detected in the brain, eye and liver. Overall, 17.0% (51/300) of the examined tissue samples were PCR‐positive. It is crucial to emphasise that these findings represent tissue‐level detection. This distinction is vital for accurate epidemiological interpretation, as it reflects the distribution of parasite DNA across specific organs rather than a simple bird‐level prevalence.

Among the positive samples, *T. canis* was the predominant species (68.6%), followed by mixed *T. canis*/*T. cati* detections (17.7%) and *T. cati* alone (13.7%). The predominance of *T. canis* suggests that canine‐associated environmental contamination is the primary driver of avian exposure in this region. However, the presence of *T. cati* and mixed detections indicates a complex transmission dynamic involving both definitive hosts. This pattern reflects a shared environmental interface where synanthropic birds, stray or owned carnivores and contaminated soil coexist (Eslahi et al. 2020; Shokri et al. 2022; Zibaei et al. 2017).

A significant finding was the marked variation in positivity among bird species. Sparrows exhibited the highest tissue‐level positivity (23.3%), significantly surpassing pigeons (6.3%) and chickens (8.8%). In sparrows, the highest detection rates were observed in the eye (31.7%) and liver (26.7%), with a statistically significant difference compared to the brain (11.7%, *p* = 0.026). Such differences were not observed in pigeons or chickens. The higher burden in sparrows likely stems from their ground‐foraging behaviour and close proximity to human dwellings (synanthropic nature), which increases their contact with soil contaminated by embryonated eggs. Consequently, sparrows may serve as effective ecological sentinels for monitoring environmental *Toxocara* contamination (Despommier 2003; von Sohsten et al. 2020).

The relatively low detection rate in brain tissue observed in the present study is noteworthy, particularly because it contrasts with the marked neurotropism commonly described in mammalian paratenic hosts such as rodents, where larvae preferentially migrate to the central nervous system during the chronic phase (Healy et al. 2022; Strube et al. 2013). This lower frequency of detection in avian brain tissue may be associated with physiological differences in the avian blood‐brain barrier, stronger immune‐mediated protection within neural tissues, or the elevated core body temperature of birds, which could influence larval migration patterns. The high detection rate in the liver is biologically plausible, as the liver is the first major organ reached by larvae through the portal circulation following penetration of the intestinal wall (Kim et al. 2024; Strube et al. 2013). Likewise, the substantial rate of detection in the eye (up to 31.7% in sparrows) suggests that ocular tropism may also be important in avian hosts. From both public health and ecological perspectives, the high larval burden in the liver is of particular concern, since consumption of raw or undercooked avian giblets may provide a potential route of transmission to humans (Stürchler et al. 1990; Taira et al. 2004). Moreover, ocular and visceral involvement may reduce host fitness, thereby increasing the likelihood of predation by definitive hosts (Mendoza Roldan and Otranto 2023; Seppälä et al. 2008).

The predominance of *T. canis* in the present study is also consistent with previous evidence indicating widespread environmental contamination with *Toxocara* eggs in different parts of Iran, including northeastern regions (Berenji et al. 2015; Ghomashlooyan et al. 2015; Maleki et al. 2018; Mohaghegh et al. 2021; Rezaiemanesh et al. 2019). Birds that forage on contaminated soil or in environments shared with dogs and cats may ingest embryonated eggs and subsequently harbour larvae in internal organs. The current findings therefore complement soil‐based surveys by showing that free‐ranging birds may not only reflect environmental contamination but may also participate in maintaining the transmission cycle indirectly, for example, through predation or scavenging by carnivores (Healy et al. 2022; Shokri et al. 2022; von Sohsten et al. 2020).

The use of multiplex nested PCR represents a major strength of this study. Conventional parasitological approaches may underestimate tissue infection because larval burdens are often low and unevenly distributed, and larval morphology alone does not allow reliable differentiation between *T. canis* and *T. cati*. The molecular approach used here therefore improves both analytical sensitivity and species‐level specificity, allowing a more refined interpretation of transmission patterns in the study area (Ding et al. 2026; Wang et al. 2018).

The spatial findings suggest that *Toxocara* positivity in birds is heterogeneous at the local level but does not form a significant global clustering pattern across the province. The visual hotspots observed on KDE likely reflect local concentrations of positive samples rather than a strong regional spatial structure. Such local variation may be influenced by microenvironmental contamination, host ecology, bird foraging behaviour and the distribution of dogs and cats (Keegan et al. 2025; Öztürk et al. 2026; Rezaiemanesh et al. 2019; Xu and Han 2024). The non‐significant Moran's I result may also be partly related to limited positive observations and the scale of analysis. Therefore, while the present data support spatial heterogeneity in exposure, they do not provide evidence for statistically significant province‐wide clustering. Future studies incorporating local hotspot statistics and environmental covariates are warranted.

Nevertheless, several limitations should be acknowledged. PCR positivity indicates the presence of *Toxocara* DNA but does not confirm larval viability; therefore, the findings should be interpreted as molecular evidence of exposure and tissue invasion rather than definitive proof of viable infective larvae. Moreover, the qualitative PCR assay did not allow estimation of parasite DNA load or larval burden. The opportunistic sampling design may have introduced selection bias, and the relatively small and uneven sample size across host groups may limit the generalisability of the findings and the statistical power for interspecies comparisons. In addition, differences in specimen sources, with sparrows and pigeons collected as field carcasses and chickens sampled after routine slaughter, may affect direct comparability among hosts. Although both bird‐level and tissue‐level positivity were assessed and within‐bird correlation was accounted for, infection intensity at the individual‐bird level could not be estimated. Furthermore, only liver, brain and eye tissues were examined; therefore, larval distribution in other organs, particularly skeletal muscles, may have been missed. Finally, the spatial analyses should be considered exploratory. KDE reflected the density of detected positive samples rather than true infection risk, as sampling effort was not incorporated. The limited number of positive locations and use of a global autocorrelation statistic may also have reduced the ability to detect localised clustering. Ecological variables such as dog and cat density, soil contamination, seasonality and climatic factors were not included. Future studies incorporating systematic sampling, larval recovery, histopathology, quantitative molecular approaches, soil contamination assessment and more detailed spatial ecological analyses would help to better clarify the epidemiological role of avian paratenic hosts in *Toxocara* transmission.

## 5 Conclusion

This study provides molecular evidence that synanthropic and domestic birds in Razavi Khorasan Province support the role of birds as potential paratenic hosts in the ecology of *Toxocara* spp. The predominance of *T. canis*, together with the detection of *T. cati* and mixed infections, suggests overlapping canine and feline sources of environmental contamination. Higher tissue‐level positivity in sparrows indicates their potential value as sentinels of environmental exposure. The detection of parasite DNA in multiple organs confirms the ability of *Toxocara* larvae to disseminate in avian hosts. Although positive cases showed local heterogeneity, no significant global spatial clustering was observed. Overall, the findings highlight the epidemiological role of birds in maintaining *Toxocara* transmission in northeastern Iran and support the need for a One Health approach to surveillance and control.

## Author Contributions

**Mohammad Reza Rezaiemanesh**: writing – review and editing, writing – original draft, conceptualization, supervision, methodology, data curation, formal analysis, validation. **Rasoul Alimi**: writing – review and editing, visualization, validation, software, formal analysis. **Abdolmajid Gholizadeh**: writing – review and editing, methodology, data curation, formal analysis. **Seyed Hossein Hejaz**: writing – review and editing, writing – original draft, conceptualization, supervision, visualization, formal analysis, funding acquisition. **Mohammad Ali Mohaghegh**: writing – review and editing, writing – original draft, conceptualization, supervision, visualization, validation, formal analysis.

## Ethics Statement

The ethical considerations of this study were approved by the Ethics Committee of the Isfahan University of Medical Sciences (IR.MUI.REC.1396.3.645).

## Conflicts of Interest

The authors declare no conflicts of interest.

## Data Availability

The data that support the findings of this study are available from the corresponding author upon reasonable request.
